# Polyprenyl Immunostimulant Treatment of Cats with Presumptive Non-Effusive Feline Infectious Peritonitis In a Field Study

**DOI:** 10.3389/fvets.2017.00007

**Published:** 2017-02-14

**Authors:** Alfred M. Legendre, Tanya Kuritz, Gina Galyon, Vivian M. Baylor, Robert Eric Heidel

**Affiliations:** ^1^Small Animal Clinical Sciences, College of Veterinary Medicine, University of Tennessee, Knoxville, TN, USA; ^2^Sass & Sass, Inc., Oak Ridge, TN, USA; ^3^Independent Consultant, Oak Ridge, TN, USA; ^4^Graduate School of Medicine, University of Tennessee, Knoxville, TN, USA

**Keywords:** feline infectious peritonitis, Polyprenyl Immunostimulant, increased survival, chronic disease, feline coronavirus, field study

## Abstract

Feline infectious peritonitis (FIP) is a fatal disease with no clinically effective treatment. This field study evaluated treatment with Polyprenyl Immunostimulant (PI) in cats with the non-effusive form of FIP. Because immune suppression is a major component in the pathology of FIP, we hypothesized that treatment with an immune system stimulant would increase survival times of cats with dry FIP. Sixty cats, diagnosed with dry FIP by primary care and specialist veterinarians and meeting the acceptance criteria, were treated with PI without intentional selection of less severe cases. The survival time from the start of PI treatment in cats diagnosed with dry FIP showed that of the 60 cats with dry FIP treated with PI, 8 survived over 200 days, and 4 of 60 survived over 300 days. A literature search identified 59 cats with non-effusive or dry FIP; no cat with only dry FIP lived longer than 200 days. Veterinarians of cats treated with PI that survived over 30 days reported improvements in clinical signs and behavior. The survival times in our study were significantly longer in cats who were not treated with corticosteroids concurrently with PI. While not a cure, PI shows promise in the treatment of dry form FIP, but a controlled study will be needed to verify the benefit.

## Introduction

Feline infectious peritonitis (FIP) is considered to be one of the most devastating diseases of domestic cats with an incidence of 2–12% (1) in multi-cat environments. FIP has long been considered fatal (2–4) and a leading cause of mortality in young cats. No clinically effective treatments exist for FIP (5). FIP has an effusive form with abdominal and thoracic fluid accumulations; a median survival time of 9 days was noted in 21 cats with effusive FIP (6). Dry (non-effusive) FIP is often characterized by pyogranulomatous infiltrates in the liver, kidneys, lymph nodes, eyes, and central nervous system. The dry form of FIP has longer survival times within a range of 1–200 days noted in 59 cats (6–13). Two cats with mixed dry and wet forms FIP treated with combinations of corticosteroids, human alpha interferon, and nelfinavir survived 181 and 477 days (7).

Mutation of the enteric coronavirus that induces a tropism for macrophages initiates the disease process (3, 14). Cell-mediated immunosuppression due to a decrease in CD4+ lymphocytes is commonly seen in cats with FIP (14). Deficiencies in cell-mediated immunity promote an exuberant production of antibodies to the coronavirus, which results in deposition of immune complexes. With immunosuppression being a major component of pathophysiology, treatment with an immune stimulant is a rational approach.

Polyprenyl Immunostimulant (PI) is a veterinary biologic licensed by the U. S. Department of Agriculture for the reduction of the severity of signs of feline herpesvirus and is safe in cats over 8 weeks of age. It was used in our pilot study to treat cats with the dry form of FIP and produced promising results (15). It upregulates Th-1 type pathway *via* toll-like receptors (16) and may thus be of benefit in the diseases involving suppression of cellular immunity. In this field study, we tried to determine if PI treatment increases survival time and quality of life in cats diagnosed with dry FIP.

## Materials and Methods

### General Study Design

The field study had a single treatment arm, without a placebo control group, and was limited to cats with non-effusive or dry FIP. Only cats in the United States and Canada were accepted. Cats were diagnosed and treated by their primary care veterinarians in conjunction with, in many cases, veterinary specialists. The veterinarians’ usual laboratories performed diagnostic tests. The study measured survival times from the start of PI treatment to death or euthanasia in terminal condition. The survival data from this study were compared to the historic data from a number of published articles. The study included cats of all signalments with clinical signs that represented the clinical spectrum of dry form FIP and were accepted and treated regardless of the severity of the disease or current treatments. Addition of appetite stimulants, antiemetics, antibiotics, vitamins, or special diets was not prohibited in our study, but the protocol advised against the use of corticosteroids because they cause immunosuppression. This study was carried out in accordance with the recommendations of the University of Tennessee Office of Laboratory Animal Care. The protocol was approved by the University of Tennessee Institutional Animal Care and Use Committee Protocol #1946.

### Case Recruitment

**Figure 1:**
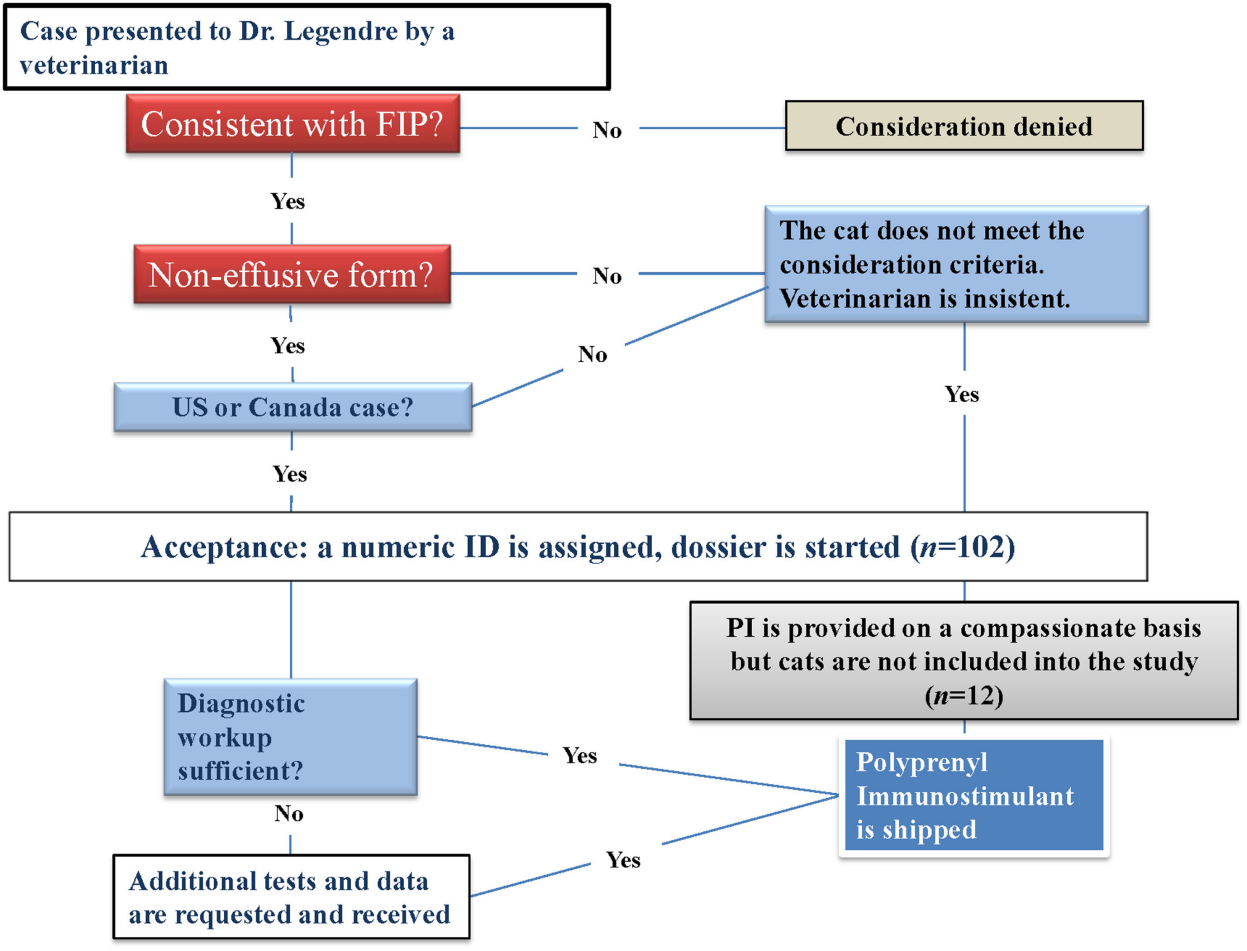
**Study decision-making tree at a glance**.

Preliminary findings of a prior pilot study were published in 2009 (15). The Veterinary Information Network site published a note of this trial in February 2010. Following the publication of the article and the note, practicing veterinarians with suspected or confirmed FIP cases contacted the Principal Investigator (AML) *via* e-mail or phone. All cats diagnosed with dry FIP by their veterinarian were considered and assigned a number (Figure 1). The initial diagnostics were done by veterinarians and reviewed and assessed for acceptance by AML based on sufficient data to support the diagnosis. In eight instances, AML accepted the cats into the study without all laboratory diagnostics if the diagnosis was made by invasive techniques [immunohistochemistry (IHC), histopathology of biopsied material, or cytology of aspirates].

The initial veterinarian diagnosis was supported using the diagnostic approach proposed by Addie et al. (2) as reflected by our data collection form (Table 1), which included questions about patient age, history, environment, and observations such as pyrexia, weight loss, lethargy, anorexia, presence of abdominal lesions (masses, enlarged mesenteric lymph nodes). Required laboratory tests included complete blood count, biochemistry, and antibody titers to feline coronavirus (FCoV) and to pathogens that may mimic FIP. Surgical biopsy or aspiration of suspected lesions was encouraged. Necropsy was offered at the University of Tennessee free of charge or veterinarians could provide results of necropsy done by their providers.

**Table 1:** **Data collection form**.

ID #			Date Polyprenyl Immunostimulant (PI) shipped							
Breed			Multi cat?				Origin			
Age now			Age at diagnosis							
Type of feline infectious peritonitis			Changes?				Histopathology?			
Date of Diagnosis			Date of PI start		Feline coronavirus (FCoV) titer			Necropsy		

	Baseline	Follow-up								
Date		30 days	60 days	90 days	120 days	150 days	180 days	210 days		
Weight										
FCoV titer										
Total protein										
Globulins										
Albumin										
HCT										
WBC										
Neutrophils										
Lymphocytes										
ALT										
Temperature										
Uveitis										
Neuro										
Diarrhea										
Abdominal mass										
Steroids										

Survival, days (check)										
Date of death										

The accepted cats received PI at 3 mg/kg orally three times per week and were clinically assessed by their veterinarians initially and then on follow-up examinations (monthly was recommended), which collected the data for the analysis as shown in Table 2. Submission of the initial and follow-up laboratory test results was required. Refills of PI were shipped to collaborating veterinarians after the results of the follow-up evaluations were received. Monitoring of the progress of the cats accepted into the study was continued until death or euthanasia.

**Table 2:** **Data analysis form**.

Case #/Name				
Breed				
Male/female				
Neutered				
Age				
Weight		Did not start Polyprenyl Immunostimulant (PI) (check) 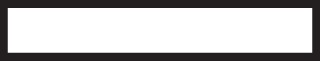		

**Clinical signs**	**1 or 0**	**1 = yes** **0 = no**	**Diagnosis**	**1 or 0**
Ocular			Abdominal imaging	
Neuro			Surgery	
Decreased appetite			Histopathology	
Fever 102.5+			Cytology	
Lethargy/depression			Elevated globulins	
Abdominal lesions			Elevated total protein	
Weight loss			Decreased albumin	
			Feline coronavirus titer	
Weight loss	1 or 0 compared to start		1:400	
@ 30 days			1:800	
@ 60 days			1:1,600+	
@ 90 days			Elevated WBC	
@ 120 days			Decreased HCT	
@ 150 days			Elevated neutrophils	
			Decreased neutrophils	
Weight gain	1 or 0 compared to start		Elevated lymphocytes	
@ 30 days			Decreased lymphocytes	
@ 60 days			Palpable/visible abdominal mass	
@ 90 days			Enlarged mesenteric lymph nodes	
@ 120 days			Colon involvement	
@ 150 days				

Weight, kg				
@ 30 days				
@ 60 days				
@ 90 days				
@ 120 days				
@ 150 days				
Status/survival			Treatment	
@ 30 days			PI	
@ 60 days			Steroids at start of PI	
@ 90 days			Steroids at 1 month after start of PI	
@ 120 days			Steroids at 2 months after start of PI	
@ 150 days			Other	
>150 days			Other	
Other				
Lost to follow-up				
			Survived after PI start	days
Improvement (subjective owner/vet assessment)				
@ 30 days				
@ 60 days				
@ 90 days				
@ 120 days				
@ 150 days				
>150 days				

Veterinarians were advised to taper corticosteroid treatments if started before the study, but they were allowed to continue corticosteroid therapy at the lowest effective dose to maintain appetite and well-being.

Quality of life assessment was done using responses on the questionnaires and communications by the primary care veterinarians and owners. In many cases, more extensive comments were recorded in the cat’s medical records, and all records were analyzed for the comments. We considered communications by veterinarians and owners regarding restoration of routines, activity levels, appetite improvement, etc. as indicators of the quality of life. Table 3 shows the questionnaire used to provide the data collected in the study.

**Table 3:** **Questionnaire for collection of patient information from the initial and follow-up examinations**.

Physical exam findings				
Date of exam			Cat’s name	
			Owner’s name	
Initial exam or recheck exam (mark)				
Date of first dose of Polyprenyl Immunostimulant (PI)				
Current dosing schedule				
Please answer the following questions based on the current examination. If you check YES, give additional details including locations, duration, and severity as necessary				
			Comments	
Weight loss	Yes	No	Current weight:	
Weakness	Yes	No		
Appetite	Yes	No		
Vomiting	Yes	No		
Diarrhea	Yes	No		
Fever	Yes	No		
Lameness	Yes	No		
Neurologic signs	Yes	No		
Paraplegia	Yes	No		
Ocular signs	Yes	No		
Other	Yes	No		
Initial history/history since last exam				

**PI Study** **Concomitant medications**				
Start date	End date	Drug	Dosage	Route
			
			

*The questionnaires were submitted with results of laboratory tests*.

### Statistical Analysis of Study Results

Skewness and kurtosis statistics found non-normal distributions for all temporal variables associated with survival. Therefore, non-parametric statistics were employed to yield inferences based on the respective research questions. Between-subjects comparisons for age groups and disease groups were conducted using Kruskal–Wallis and Mann–Whitney *U*-tests. In addition to means and SD, medians and interquartile ranges were reported to give context to non-parametric statistical findings. Kaplan–Meier survival curves were used to display the cumulative survival of cats across time. *t*-tests were employed for the comparison of mean and SD. An alpha value of 0.05 assumed statistical significance, and all analyses were conducted using SPSS Version 21 (Armonk, NY, USA: IBM Corp.).

## Results

### Consideration and Acceptance

Consideration began March 1, 2010, and ended May 6, 2011. A total of 102 cats were considered for the study. All cats were diagnosed by their primary care veterinarians. There were 60 cats that met the qualification criteria and were therefore accepted into the study, and 23 of those had been referred to specialists, including veterinary ophthalmologists (12), internists (6), neurologists (3), ophthalmologist and a neurologist jointly (1), and a veterinary cardiologist (1). The remaining considered cats were disqualified before or during the study for the following reasons: insufficient diagnostic information (10), died before PI arrived (10), wet form FIP (10; PI was provided on a compassionate basis), treatment was stopped after one to two doses (2), overseas cases (2; PI was provided on a compassionate basis), incorrect diagnosis (2; PI was provided to one of the two cats on a compassionate basis), cats that were lost to follow-up because the veterinarian never provided any information after PI was shipped (5) and one cat enrolled in error, i.e., started the treatment prior to March 1, 2010 (1). The accepted cats were evaluated and treated by veterinarians throughout the USA (58), and in Canada (2) in their practices. Fewer than 32% (19 of 60) of the cats were diagnosed at 10 days or less before the start of PI treatment. The remaining 41 cats were diagnosed with dry FIP 11 or more days before the beginning of treatment with PI. The mean time span between the diagnosis and the treatment was 22.97 ± 21.60 days. The cat that died after the administration of the first dose was diagnosed 161 days before the start of PI treatment.

### Diagnostics

#### Signalment

**Figure 2:**
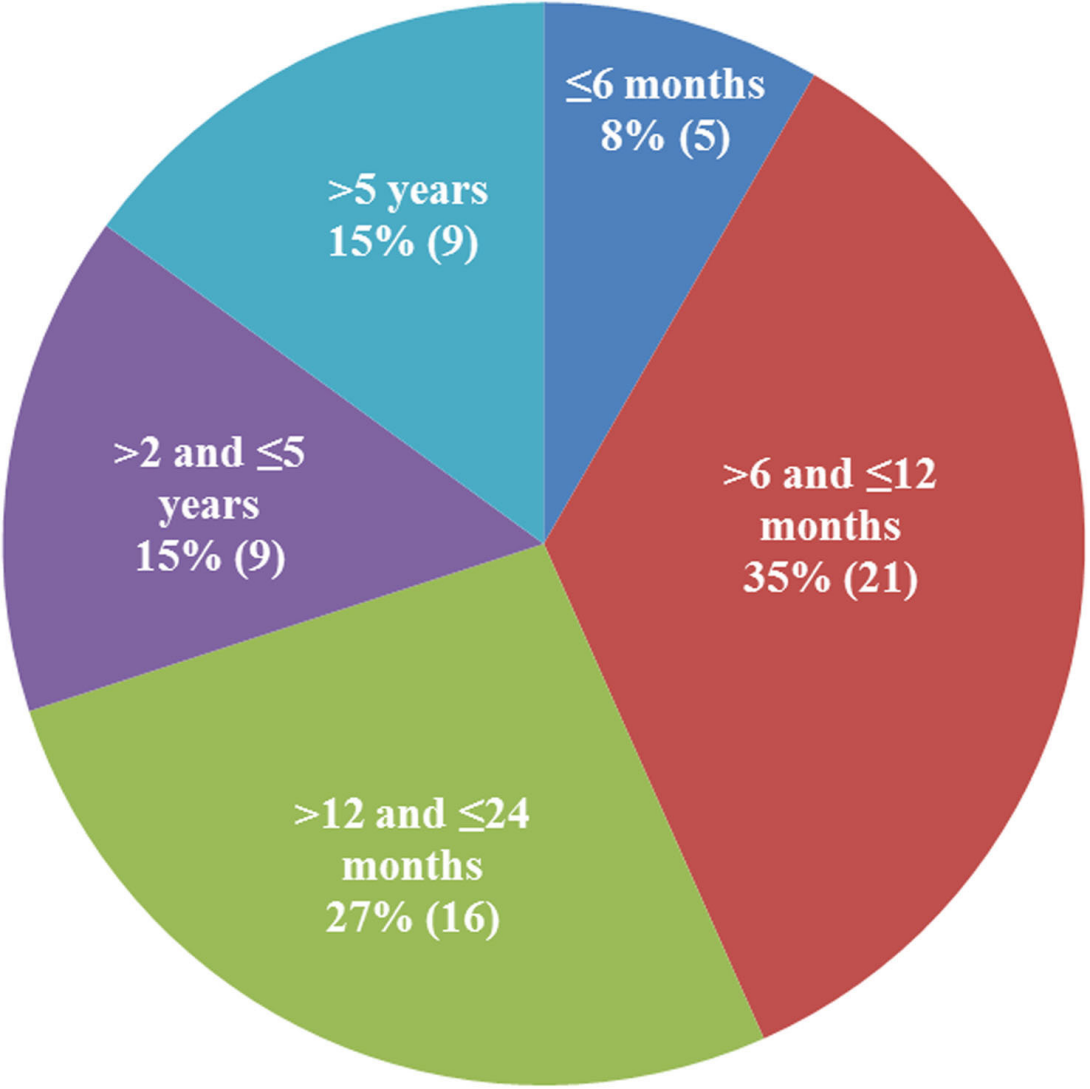
**Age distribution of the qualified patients**. The majority of cats diagnosed with dry feline infectious peritonitis were under 2 years of age (70%).

There were 25 female, 1 hermaphrodite, and 34 male cats; 38 were non-purebred and 22 were purebred (5 Ragdoll, 4 Siberian, 3 Bengal, 3 Maine Coon, 2 Siamese, 2 Sphynx, 1 Tonkinese, 1 Manx, and 1 Birman). Data on the household were provided for 30 cats; 22 of the 30 were from multi-cat households and 8 from single-cat households. The age distribution of the 60 cats is shown in Figure 2. Seventy percent of the cats were under 24 months old, and 43% were under a year of age.

#### Clinical Signs

**Figure 3:**
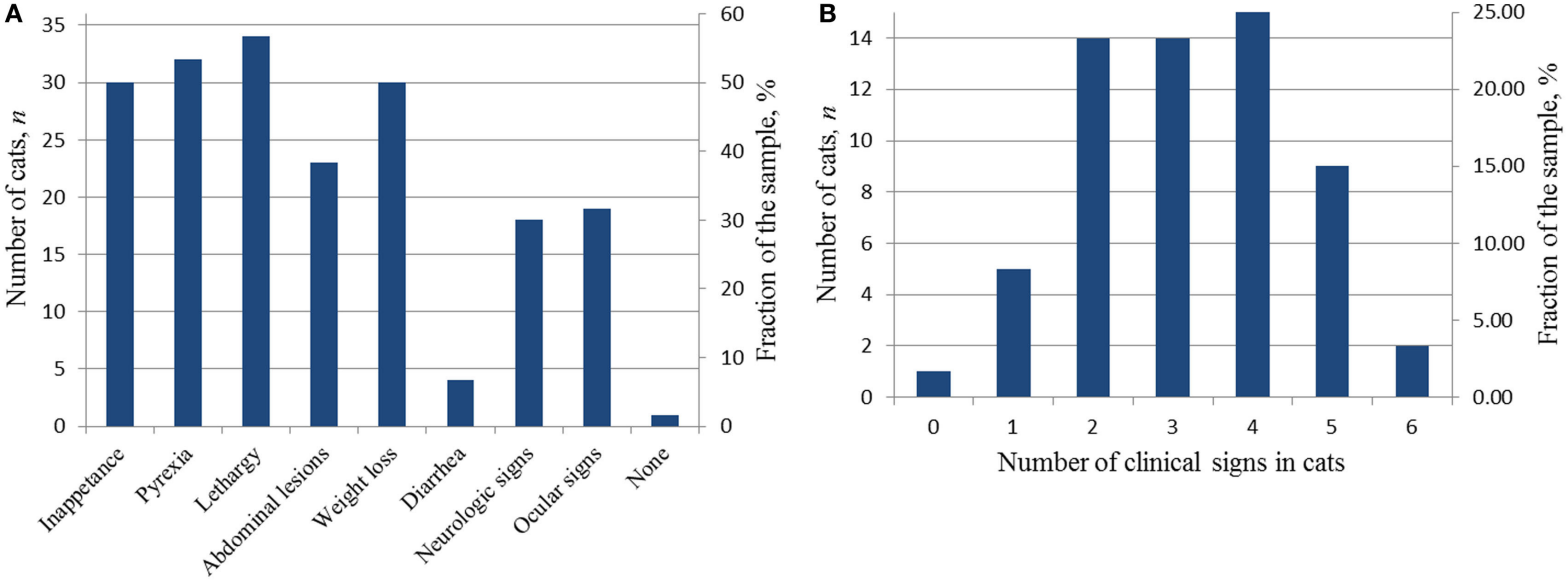
**Diagnostic clinical signs in the cats accepted into the study at the time of the initial presentation**. **(A)** Distribution of clinical signs in the study sample; **(B)** distribution of the number of clinical signs in individual cats.

Fifty-nine of the 60 cats accepted into the study had clinical signs of FIP listed on the algorithm proposed by Addie et al. (2). The presence of clinical signs caused the cats’ owners to bring the cat to their primary veterinarian for examination. During this initial examination, the clinical signs were documented in medical records. Figures 3A,B show the clinical signs and their distribution in the cats accepted into this study. Fifty-four of 59 cats showed two or more clinical signs (Figure 3B) with five cats having one sign. Of the five cats with one sign, 2/5 were neurologic, 1/5 ocular, 1/5 was evaluated because of persistent vomiting (an abdominal mass in the ileocolic region was discovered at the examination), and no data of a comprehensive initial examination were provided for one whose abdominal mass was discovered at physical examination.

One cat was included in the study that had no clinical signs when seen for neutering. This 8-month-old, male Siberian cat had a 10.7 g/dL serum total protein on a pre-anesthesia screening. The albumin was 2.1 g/dL, and the globulin was 8.6 with an A/G ratio of 0.24. The serum electrophoresis was interpreted as polyclonal gammopathy. The cat was anemic with a hematocrit of 23%. The coronavirus antibody titer was positive at 1:1,600, and the feline leukemia antigen was negative and the feline immunodeficiency antibody titer was negative. Thoracic radiographs showed an increase in cranial mediastinal density, and enlarged mesenteric lymph nodes were seen on abdominal ultrasound. Lymph node aspirates showed an increase in neutrophils on aspirate cytology.

Fifty-nine of the 60 cats on the study were categorized into one of five subforms of dry FIP based on the initial clinical signs, physical examination findings by primary veterinarians (and specialists where applicable) and diagnostic workup. One cat had no clinical signs. The subform categories were distributed as follows: mixed (18/59), gastrointestinal (16/59), non-localized (11/59), ocular (9/59), neurologic (5/59).

At the initial presentation, the cats in the gastrointestinal category had anorexia (15/16), diarrhea (4), and/or vomiting (3), which were the primary reasons for the veterinary visit. Abdominal masses were found in 23/59 cats in the study, and the cats were categorized into either the gastrointestinal or mixed subform depending on whether they had additional clinical signs more often associated with another subform.

Ocular changes were reported in 17 cats and included anterior uveitis (17/17), retinal detachment (2/17), and keratic precipitates (4/17). One cat had a corneal ulcer. In 10/17 cats, ocular signs were the reason for the initial veterinary visit, and the cats were assigned to the ocular subform. The other 7 cats with ocular signs also had neurologic, abdominal, or non-localized signs and were classified as having mixed form. Neurologic signs, such as seizures, ataxia, and disorientation, were the main reason for the veterinary evaluation in 5/59 cats, and they were categorized as neurologic subform. The “non-localized” category (11/59) included cats with persistent fever uncontrollable-with-antibiotics, lethargy, anorexia, and/or weight loss. The mixed subform cats had simultaneous signs from two or more subcategories, such as ocular combined with neurologic signs (e.g., uveitis and seizures).

#### Hematology, Serology, Differential Testing

Results of hematology tests from blood drawn during the initial examination are shown in Table 4. The blood testing data were unavailable for nine cats initially diagnosed based on histology and cytology findings consistent with FIP which were accepted as diagnostic. Two cats had serology and hematology tests done after the acceptance and the start of the treatment, and those later data are not included on the table. In some cases the testing did not include items of interest, thus the data sets are not complete.

**Table 4:** **Hematology test results at the first presentation considered in the feline infectious peritonitis diagnosis**.

Measurement	Reference interval	Data sets received, *n*	Mean	WNL, *n* (%)	Below normal, *n* (%)	Above normal, *n* (%)
Albumin, g/dL	2.3–3.9	51	2.5 ± 0.5	32/51 (62.7)	19/51 (37.3)	0
Total protein, g/dL	5.9–8.5	51	9.8 ± 1.5	12/51 (23.5)	0	39/51 (76.5)
Globulins, g/dL	3.0–6.6	51	7.3 ± 1.6	18/51 (35.3)	0	33/51 (64.7)
A/G ratio	0.4–0.8	50^a^	0.37 ± 0.14	See the breakdown below:		
	<0.8			2/50 (4.0)^b^	48/50 (96.0)	0
	<0.6				36/50 (72.0)	N/A
	<0.4				32/50 (64.0)	N/A
Total bilirubin, mg/dL	0.0–0.4	50	0.5 ± 1.1	42/50 (84.0)	N/A	8/50 (16.0)
WBC, 10^3^/μL	4.2–15.6	51	15.6 ± 10.6	27/51 (52.9)	1/51 (2.0)	23/51 (45.1)
HCT, %	29–45	49	29.4 ± 6.9	20/49 (40.8)	28/49 (57.1)	1/49 (2.0)
Neutrophils	Varies	44	N/A	19/44 (43.2)	1/44 (2.3)	24/44 (54.5)
Lymphocytes	Varies	48	N/A	32/48 (66.7)	16/48 (33.3)	0

*Seven animals were accepted based on the results of histopathology and cytology, and hematology results for two cats were received after the start of the treatment*.

*^a^In one cat, concentrations of individual fractions were provided in lieu of the total globulin level and showed markedly elevated serum globulins and gammopathy. In another cat, no albumin levels were provided*.

*^b^A/G ratio was 0.8 in two cats; no cat had A/G ratio above 0.8*.

*WNL, within normal limits*.

Jaundice was observed in one cat whereas hyperbilirubinemia was noted in 8/50 cats. Anemia (HCT < 29%) was observed in 28 of 49 cats. Increased WBC counts were noted in 20/50, neutrophilia was observed in 24/44, and lymphopenia in 16/48 cats.

Hyperglobulinemia and/or an albumin/globulin (A/G) ratio ≤0.6 were noted in 48 of the 50 (96%) cats; two cats had A/G ratio equal to 0.8. The mean and SD of the value in the whole group was 0.37 ± 0.14, and the spread is shown in Table 4.

The antibody titers were tested by IFA in 49 cats and, on the scale proposed by Addie et al. (17), were ranked from high positive (400–1,280; *n* = 13) to very high positive (>1,280, *n* = 36); 10 of the 49 had titers > 12,800. In the two cats with low positive titers (100), the diagnosis was confirmed by immunostaining of biopsied lesions for the FCoV antigen. The 7b ELISA was used in three cats. The 3c PCR test done in three cats and showed negative results in two; these two cats were retested by IFA with positive results and also had the diagnosis by cytology.

We collected data sets on differential testing on 50 cats; the tests included FeLV antigen (45), feline immunodeficiency virus (FIV) (45), and toxoplasma (18). Except in one cat who was FIV positive, all other test results were negative. No serologic data were available for 10 cats. In the group with probable diagnosis with four missing data sets, one cat was diagnosed by neurologist based on the results of spinal tap and MRI, and another one had uveitis as a 3.3 lb 7-month Maine Coon kitten. In the group with the diagnosis confirmed by histologic, cytologic, and immunochemical methods, of the six cats without differential data sets, four were diagnosed by IHC and two by histology on the biopsied tissues.

#### Specialized Laboratory Testing

Specialized laboratory testing methods used to confirm the dry FIP diagnosis are summarized in Table 5. Histology and cytology were performed in 36 cats and were conclusive in 34, they were further validated by immunostaining for FCoV antigen in 13/36 cats. One cat with pyogranulomatous mesenteric lymphadenopathy on lymph node aspirate also had elevated FCoV transcripts in the RT-PCR test on the same sample.

**Table 5:** **Specialized tests used in support of the diagnosis**.

Test type	Other tests on the same cat	Total tests, *n*		Total histology, cytology, necropsy, *n*	Total CSF tap, *n*	Total ocular centesis, *n*	Total imaging, *n*
		By test	Total				
Cytology on fine needle aspirate		1^a^	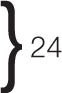	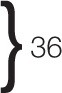			
Histology	Only	8					
	+IHC	12					
	+Necropsy	2					
	+IHC+necropsy	1					
Necropsy	Only	12					
CSF tap cytology	Only	1			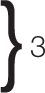		
	+MRI	2					
Q-PCR (m-gene mRNA) on aqueous humor		1				1	
Thoracic and abdominal imaging (X-rays and ultrasound)	Only	3						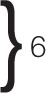
	+Histology or cytology or necropsy	3^c^						
Consistent with FIP				34/36	3/3	1/1	3/3^c^
Inconclusive				1/25^a^	0	0	0
Inconsistent with FIP				1/25^b^	0	0	0

*^a^Low cellularity*.

*^b^Necropsy results inconsistent with FIP; details are provided in the text*.

*^c^The count is included into histology, cytology, necropsy total (*n* = 36)*.

Three cats with neurologic disease had the diagnosis confirmed by CSF tap, and two of those had MRI results consistent with the FIP diagnosis. For one cat with ocular form ocular centesis followed by quantitative PCR confirmed the presence of high titers of FCoV subgenomic mRNA of the M gene.

Necropsy was done of 15/60 cats. In 3/15, the necropsy was done of cats whose diagnosis was previously confirmed antemortem by histology or cytology. Histopathological analysis of the necropsied tissues was conclusive for FIP for 14/15 cats and inconclusive for the 965-day survivor.

#### Concurrent Treatments Used in the Study

Of the 60 cats accepted into the field study, 13 received PI as the only treatment; the other 45 cats received treatments before the enrollment and/or concurrently with the PI including one or more of appetite stimulants, antiemetics, antibiotics, corticosteroids, vitamins, and/or special diets. There were no data on concurrent treatments for two cats.

Sixty-two percent of the cats (36/60) were prescribed corticosteroids orally at the time of the initial diagnosis (27/36), topically (ocular, 7/36), or both (2/36). In 4/36 cats; the corticosteroid treatment was stopped before or shortly after beginning the treatment with PI. Statistics on the use of corticosteroids are presented in Table 6. During the study, 31 cats received corticosteroids concurrently with PI (7 ocular topical and 25 systemic or systemic with ocular topical), and 27 cats were treated with PI without concurrent corticosteroids.

**Table 6:** **Survival time by the subform of the disease. No statistically significant differences were observed between any groups**.

Form	Treatment	*n*	Survival, days mean ± SD	Survival range, days
Ocular	PI	3	99.33 ± 109.77	32–226
	PI + ToCS	2	185.50 ± 159.10	73–298
	PI + SyCS	4	67.75 ± 80.19	6–184

Neurologic	PI + SyCS	5	38.80 ± 38.21	5–100

Gastrointestinal	PI	11^a^	252.45 ± 533.25	15–1,829
	PI + SyCS	5	53.20 ± 75.60	7–185

Non-localized	PI	6^b^	268.83 ± 363.00	4–965
	PI + ToCS	1^c^	60	60
	PI + SyCS	4	11.00 ± 13.47	7–31

Mixed	PI	8^b^	77.38 ± 94.87	1–131
	PI + ToCS	6	32.67 ± 28.49	4–39
	PI + SyCS	4	17.25 ± 16.46	6–77

No signs	PI	1	148	148

*PI, treated with Polyprenyl Immunostimulant, no concurrent corticosteroid treatment; PI + SyCS, treated with Polyprenyl Immunostimulant and systemic corticosteroids concurrently (includes 1 combined systemic and topical ocular corticosteroid treatment); PI + ToCS, treated with Polyprenyl Immunostimulant and topical ocular corticosteroids concurrently*.

*^a^Includes two cats whose corticosteroid treatment was stopped at the start of PI treatment*.

*^b^Each number includes one cat whose corticosteroid treatment was stopped at the start of PI treatment*.

*^c^The cat initially diagnosed with non-localized form started ocular topical corticosteroid treatment after uveitis developed*.

The four cats whose corticosteroid treatment was stopped in compliance with the requested test protocol before or at the start of PI treatment all had clinical signs consistent with FIP: weight loss (4/4), ataxia (1/4), abdominal masses (3/4), pyrexia (2/4), lethargy (2/4), anorexia (2/4) with the number of clinical signs from 2 to 5. Their laboratory tests also supported the diagnosis.

Two veterinarians started corticosteroid treatment when the cats deteriorated, one at 9 and at one 24 days before death; these cases were end-of-life care and were not counted as corticosteroid receivers. One cat was given one dose of nelfinavir the day before dying by her veterinarian owner.

#### Duration of Survival Post-Diagnosis and Clinical Progress

Duration of survival was determined as the time from the start of the PI treatment to death or euthanasia. Of the 60 cats treated with PI, 16 survived for over 100 days, 8 cats survived for over 200 days, 4 cats survived for over 300 days (one additional cat survived for 298 days and is not counted here), 2 for over 900 days, and 1 cat for 1,829 days.

**Figure 4:**
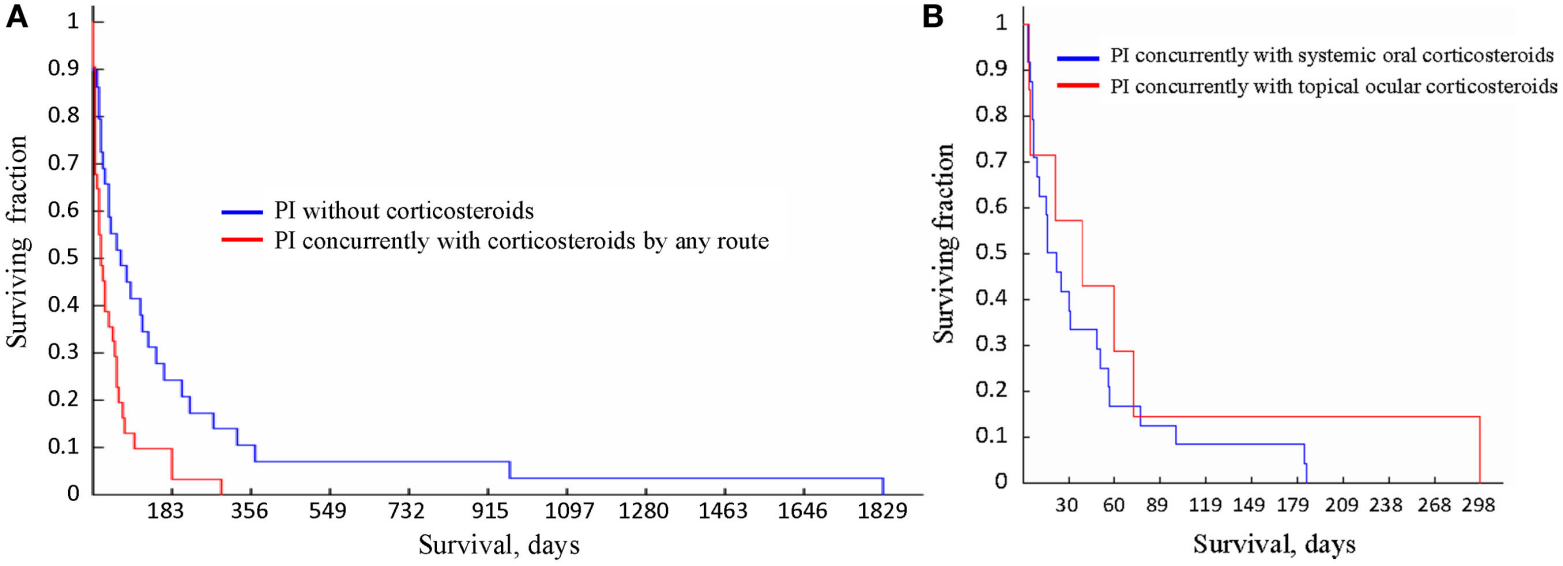
**Survival curves for the cats receiving different treatments**. **(A)** Survival of the cats treated with Polyprenyl Immunostimulant (PI) without concurrent corticosteroids (blue) was significantly longer (*p* = 0.003, Mann–Whitney *U*-test) than of the cats treated PI with corticosteroid administered concurrently by any route (red). **(B)** Survival of the cats treated with PI with concurrent corticosteroid administration topically (red) or orally (blue) did not differ significantly (*p* = 0.57, Mann–Whitney *U*-test).

The survival times of the cats in the three groups, i.e., (1) treated with oral corticosteroids concurrently with PI, (2) treated with topical ocular corticosteroids concurrently with PI; and (3) treated with PI without concurrent use of corticosteroids were significantly different from each other (*p* = 0.03, Kruskal–Wallis test). Table 7 lists all statistical data, and the survival curves are presented in Figure 4. For cats who did not receive corticosteroids the median survival time was 73.5 days (*n* = 27). For the cats that received corticosteroids by any route concurrently with PI, the median survival time was 21.5 days (*n* = 31). The difference in survival times between the groups (corticosteroid receiver versus non-receiver) was significant (*p* = 0.003, Mann–Whitney *U*-test). There was no significant difference in the survival times of the cats treated with corticosteroids systemically or topically (*p* = 0.57, Mann–Whitney *U*-test). The four cats whose initial corticosteroid treatment was stopped before the treatment with PI survived for 79, 91, 279, and 1,829 days. No data on concurrent treatments were available for two cats diagnosed by histology (1) and histology with IHC (1).

**Table 7:** **Survival of cats treated with or without corticosteroids concurrently with Polyprenyl Immunostimulant by the method of the diagnosis**.

Treatment	*n*	Survival statistics, days				*p*-value (Mann–Whitney *U*-test)
		Range	Median	Mean	SD	
**All study cats (*n* = 60)**						
No concurrent corticosteroids	27	3–1,829	73.5	201.4	378.6	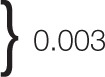
Concurrent corticosteroids:	31	3–298	21.5	47.5	49.3	
systemic	24	3–185	16	40.5	71.4	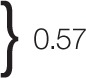
topical	7	4–298	30	51.2	103.3	
No data on concurrent treatments	2	1–15	N/A	8	9.9	N/A
**Diagnosis confirmed by specialized tests on biopsied tissues (*n* = 34)**						
No concurrent corticosteroids	20	3–1,829	63	180.5	400.0	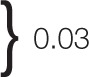
Concurrent corticosteroids	12	4–185	20.5	38.9	51.3	
No data on concurrent treatments	2	1–15	N/A	8	9.9	N/A
**Diagnosed without confirmation on biopsied tissues (*n* = 26)**						
Necropsy/cytology inconclusive, no concurrent corticosteroids (*n* = 2)	7	4–965	148	261.1	329.7	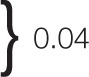
No concurrent corticosteroids (*n* = 5)						
Concurrent corticosteroids	19	3–298	22	52.8	74.2	

For the cats receiving PI alone and whose diagnosis was not confirmed by cytology, histology (ante- or post-mortem) survival times were 261.1 ± 329.7 days (4–965 days, median 148 days, *n* = 7); the cats who were treated with PI and corticosteroids concurrently with PI survived 52.8 ± 74.2 days (3–298 days, median 22 days, *n* = 19). The difference in the survival between the cats treated or untreated with corticosteroids concurrently with PI was significant (*p* = 0.03).

Cats whose diagnosis was confirmed by any type of analysis of biopsied or necropsied samples and treated with PI without concurrent corticosteroids survived 180.5 ± 400.0 days (3–1,829 days, median 63 days, *n* = 20); the cats treated with PI and corticosteroids concurrently survived 38.9 ± 51.3 days (4–185 days, median 20.5 days, *n* = 12). Two cats with inconclusive results of cytology (1) and necropsy (1) were accounted for as unconfirmed by those methods. The difference in the survival between the cats treated or untreated with corticosteroids concurrently with PI was significant (*p* = 0.04). A subgroup in which the diagnosis was also confirmed by IHC included eight cats treated with PI only (survived 8–1,829 days) and four cats treated with PI concurrently with corticosteroids (survived 7–185 days). No concurrent treatment data were available for one cat who survived one day. The size of those subgroups was insufficient to render statistical power to the analysis.

There was no significant difference in survival times between groups treated with PI only and diagnosed without biopsy-based tests (*n* = 20) versus those diagnosed with biopsy-based tests (*n* = 7; *p* = 0.27, Mann–Whitney *U*-test). Similarly, there was no difference in survival times between groups treated with corticosteroids concurrently with PI, which were diagnosed with tissue biopsies (*n* = 12) or without it (*n* = 19; *p* = 0.93 Mann–Whitney *U*-test).

No significant difference was found between the survival times for the cats receiving oral (*n* = 24) or topical ocular corticosteroids (*n* = 7, *p* = 0.57, Mann–Whitney *U*-test); and the median survival times were 16 and 30 days, respectively. No significant differences were noted between survival times of cats with different subforms of the disease regardless of the use of steroids (Table 6). Survival times of cats belonging to age groups under 6, 7–12, 13–24 months, and over 25 months did not differ significantly (*p* = 0.90, Kruskal–Wallis test).

After the beginning of treatment, the non-effusive form progressed to effusive in six cats (10%), and five of those died or were euthanized within 2 weeks thereafter. One cat (#31) whose initial corticosteroid treatment was stopped at the beginning of PI trial developed palpable abdominal masses and effusion after 3 months of the treatment, which resolved by the next monthly visit. After 6 months on the treatment, a small mass was palpated and a small amount of fluid in mid-cranial abdomen was identified per the veterinarian’s records. The mass remained unchanged for the next two monthly visits, and the cat developed a distended belly with palpable fluid by the eighth month of the PI treatment. The amount of the fluid decreased by the next monthly check. Between 1 and 8 months on the treatment, the cat was doing clinically well, gained weight, returned to normal routines, played, and had an appetite. The cat started declining in the ninth month of the PI treatment, lost weight, and there was an increased amount of abdominal fluid, and the cat died naturally after 279 days from the start of the treatment.

Three of the 17 cats with ocular disease survived for over 180 days. Their initial signs included anterior uveitis (3), keratic precipitate (1), discoloration (1), anisocortia (1). In two of those cats the anterior uveitis was significantly improved or resolved after 2 months on PI treatment with no corticosteroids. The uveitis did not improve in the one cat who was receiving topical ocular corticosteroids concurrently with PI, and the eyes were enucleated.

In the 13/22 cats with palpable abdominal masses that survived over 30 days (life span 57–1,829 days), the reduction or resolution of the abdominal masses was noted in 6/13 during the first or second monthly follow-up examinations, three of the six cats received corticosteroids together with PI. One of the six cats (#31, described above), whose initial neurologic signs resolved and palpable masses were no longer reported after 1 month of the treatment, redeveloped a small palpable abdominal mass and abdominal fluid at 6 months into the treatment and 3 months prior to her natural death at 279 days; the corticosteroid treatment of this cat was stopped before PI treatment began. In another cat who received no corticosteroids, the masses were initially resected and did not reappear until 1 month prior to euthanasia at 374 days. The masses remained unchanged on palpation in 4/13 cats; all four cats received no corticosteroid treatment. The findings were confirmed by ultrasound tests in one of those four, two cats died before the follow-up examination, and follow-up data were not available for one cat.

**Figure 5:**
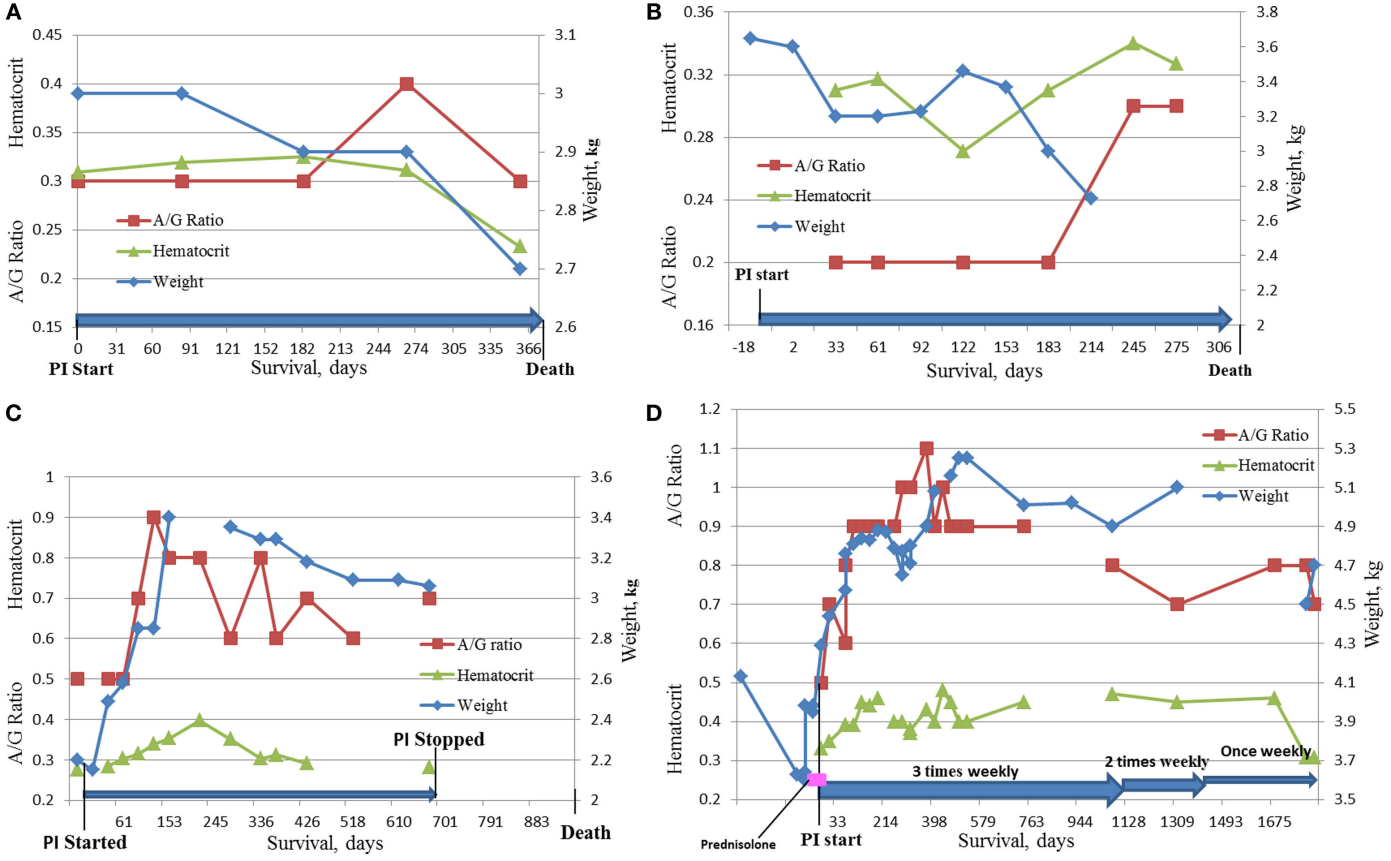
**Dynamics of HCT, A/G ratio, and weight in the four longest survivors on the study**. **(A)** Cat #2, 375 days survival; **(B)** Cat # 52, 334 days survival; **(C)** Cat #78, 965 days survival; **(D)** Cat #105, 1,829 days survival.

Four cats survived over 300 days and were considered long term survivors. Their records were scrutinized in considerable detail after death. The summary of the data for these cats is presented in Table 8 and Figure 5. All four cats were brought initially to their veterinarians with signs typical of dry FIP, including inappetance (3/4), lethargy (3/4), and weight loss (4/4). One cat had persistent diarrhea and vomiting; in two of the four cats, abdominal masses were detected by their veterinarians. The A/G ratios for all four cats ranged from 0.3 to 0.5. Three of the four cats were anemic, and all had moderate to high coronavirus antibody titers of 1:320 (K-ELISA), 1:800, 1:1,600 and 1:6,400 (all three by IFA). In two of the four cats, histopathology of biopsied tissues had pyogranulomatous inflammation, and the diagnosis was validated by immunostaining for FCoV antigen. Cat 105, the longest survivor (1,829 days), initially received prednisolone to control the weight loss but the corticosteroid therapy was discontinued at the start of PI treatment. Based on reports by owners and veterinarians, all four cats returned to normal behavior by the first checkup visit, about 1 month from the beginning of the treatment, and the weight either stabilized or increased. The clinical signs of vomiting, diarrhea, lethargy, etc. also resolved. Three cats lost weight before death; data were unavailable for one. The A/G ratios increased after 2 months on the PI treatment in the two longest survivors, and reached >0.8, and anemia improved in one and resolved in another one. The borderline anemia, although somewhat improved, was present in the Cats 2 and 52 survivors and became more pronounced before death. The treatment was stopped after about 700 days in Cat 78, and no laboratory test data are available from that time until his death at 965 days when necropsy was performed. The PI treatment frequency was tapered first to twice weekly and then to once weekly in Cat 105. All cats were euthanized in extremis. The necropsies on the Cat 2 showed lesions consistent with FIP. The necropsy on Cat 78 showed lymphoplasmacytic interstitial nephritis and cystitis cystica without lesions of FIP. Toxicosis, azotemia, and ischemia probably associated with renal failure led to the euthanasia of Cat 105. The ultrasound showed no masses on the internal organs and the results were unremarkable; no necropsy was performed.

**Table 8:** **Case summaries for the cats with feline infectious peritonitis (FIP) surviving over 300 days on Polyprenyl Immunostimulant (PI) treatment**.

	Cat #2 (A)	Cat #52 (B)	Cat #78 (C)	Cat #105 (D)
Age at Dx	11 years	12 months	6 months	3 years

Sex	FS	FS	MN	MN

Breed	DMH	Bengal	DSH	DLH

Housing density	2 cats	>3 cats	>20 cats	>3 cats

Household and FIP history	Not known	Previously lost 3 cats to FIP	Rescued by a rescue with FIP outbreak. Foster queen died of FIP	Not known

Keeping condition	Inside	Inside	Inside	Inside

Survival time, days	**375**	**334**	**965**	**1,829**

**Initial presentation and diagnostics**				

Days between initial visit and the start of PI	36	23	19	31

Diagnostic signs	Frequent vomiting, persistent diarrhea, weight loss. Not active. Palpable abdominal mass	Weight loss, vomiting, persistent diarrhea with occasional blood streak	URI, weight loss, weakness, possible diarrhea (too many cats to tell), poor appetite	Sudden weight loss, abdominal mass noted on ultrasound

Additional clinical signs	CRF (Dx 1 month after FIP)	None	Submandibular lymphoadenopathy, gingivitis, conjunctivitis	Mild gingivitis at the end of life

Differential	FIV-neg, FeLV-neg, toxoplasma-neg	FIV-neg, FeLV-neg, toxoplasma-neg	FIV-neg, FeLV-neg, toxoplasma-neg	FIV-neg, FeLV-neg, toxoplasma-neg

Albumin, g/dL	2.3	2.6	2.6	2.3

Tp, g/dl	9.3	10.8	8.3	8.8

Globulin, g/dl	7.3	8.2	5.7	6.5

A/G ratio	0.3	0.3	0.5	0.4

Bilirubin, mg/dL	0.1	0.1	0.1	0.4

WBC cells/uL	23,400	22,600^a^	20,300	11,700

HCT, %	30.9	31.0^a^	8.4	33 (N 32–49)

Neutrophilia	YES	YES^a^	YES	NO

Lymphopenia	YES	YES^a^	YES	YES

Monocytosis	YES	NO^a^	NO	NO

Feline coronavirus (FCoV) titer	1:6,400 (ELISA IFA)	1:320 (7B ELISA)	1:1,600 (ELISA IFA)	1:800 (ELISA IFA)

FIP subtype	GI	GI	Non-localized	GI

Specialized laboratory testing and findings	Ultrasound, resection, and anastomosis of ileocecal region. Biopsies had pyogranulomatous reaction, IHC was FCoV antigen-positive	Not done	Not done	Polyclonal gammopathy, FIP mRNA-, ultrasound, FNA, biopsy. Histopathology revealed pyogranulomatous lymphoadenitis and pancreatitis. IHC positive for FCoV antigen

Concurrent medications	Calcitrol 10 mg orally daily, Sucralfate PRN, Metoclopramide PRN	Metoclopramide PRN	PI treatment stopped after about 700 days	PI was tapered to 2× weekly after 3 years and to once weekly after 4 years

**Progress on the PI treatment**				

Hyperglobulinemia and A/G ratio	No major change	No major change	Changed to WNL after 2 months	Decreased to normal range after 1 month

Anemia	No. Before death only	No. Last test 40 days before death	Hematocrit increased to >30% after 2 months on PI. No data after 700 days	Increased to >35% after 2 months on PI, decreased before death

Diagnostic clinical signs	Resolved	Resolved	Resolved	Resolved

Life quality	Improved and returned to normal	Returned to normal	Improved, stable (records until the end of PI treatment). Cystitis Dx after 600 days on PI	Returned to normal

Cause of death	Euth: weight loss, lethargic, dehydrated, trouble breathing	Euth: inappetance, lethargy, weight loss, fever	Euth *in extremis*	Euth: anorexia, vomiting, severe azotemia indicating kidney failure or severe trauma from infection or toxin/ischemic injury. No abdominal mass on ultrasound before death

Necropsy	Pleural effusion, small mesenteric mass, close to the resection site. Multifocal granulomatous colitis and hepatitis consistent with FIP	Not done	Mild changes consistent with prior hepatic injury and nephritis, cystitis cystica	Not done

*^a^Tested 9 days after the start of the PI treatment*.

*CRF, chronic renal failure, chronic renal insufficiency; Euth, euthanasia; FCoV, feline corona virus; FeLV, feline leukemia virus; FIV, feline immunodeficiency virus; FNA, fine needle aspirate; IHC, immunohistochemistry; URI, upper respiratory infection; WNL, within normal limits*.

A number of veterinarians and owners voluntarily provided information on the quality of life in communications, and veterinarians commented from the first recheck on after 30 days of PI treatment using the questionnaire. More quality-of-life-related comments were recorded by veterinarians on medical records which were analyzed. We found improved quality-of-life comments for 32 of the 34 cats that survived for more than 30 days. All comments for those 32 cats indicated an improvement in the perceived quality of life (“return to normal,” “as before the disease,” “appears healthy,” got back to normal routines, and/or improvement in appetite, mobility, socialization, and responsiveness) during the preceding period, most commonly every month on the treatment. Weight stabilized or increased in 31/32 patients, while 1/32 continued to lose weight while showing improvement in behavior and appetite.

No toxicity or adverse events due to the administration of PI were reported by the veterinarians or owners.

## Discussion

We report that of the 60 cats with presumed non-effusive FIP diagnosed using the recognized algorithm (2) and treated with PI 1 cat survived for 1,829 days, 2 cats for over 900 days, 4 cats survived over 300 days, 8 for over 200 days (one of those survived 298 days), and 16 lived over 100 days from the start of treatment. The 31 cats given oral corticosteroids or receiving topical ocular corticosteroids concurrently with PI survived a mean of 47. 5 ± 49.3 days (3–298 days, median 21.5 days), while the 27 cats treated with PI without corticosteroids survived a mean of 201.4 ± 378.6 days (3–1,829 days, median 73.5 days), which is significantly longer (Table 7; Figure 4A). Currently the most common therapy for FIP is corticosteroid (2, 4).

Of the 35 cats started on corticosteroids at the time of diagnosis, the treatment was stopped in 4 cats before the start on PI, while 31 were continued on corticosteroids concurrently with PI. The four cats whose corticosteroid treatment was stopped survived a mean of 569.50 ± 844.65 days (79–1,829 days, median 91 days). These four cats had multiple signs of the disease, and none of the four cats at the time of diagnosis appeared less severely affected than the other cats in our study.

In the subgroups of FIP, there was a difference in the likelihood of corticosteroid use, with 31% (5/16) in the GI subform of FIP receiving corticosteroids to 100% (5/5) in neurologic subforms receiving corticosteroids. Both topical and ocular corticosteroids appeared to reduce survival times when given concurrently with PI (Figure 4B).

We accepted all cats that met the inclusion criteria and did not intentionally select less severe cases for the study. No assessment of the severity of the disease was done. Our first assumption was that corticosteroids were used in the more severe cases but we could find no justification for that assumption. But the small sample size does not allow ruling out that the shorter survival times of the cats concurrently treated with PI and corticosteroids may indirectly reflect the severity of the disease.

We observed no statistically significant differences between survival of the cats either for the subforms of the disease (ocular, neurologic, gastrointestinal, non-localized, or mixed), or between different age groups (≤6 months, 7–12 month, 13–24 months, and over 25 months) which may be because of a very wide variance in each of the groups. We did not have a sufficient number of cats in each group for the statistical power to validate any conclusions.

The literature offers limited data on survival times of cats with dry form FIP with or without treatments. Cats in the literature with dry form FIP treated with corticosteroids and supportive care had a survival range of 1–200 days (*n* = 51). One early study (8) examined field records for intestinal, granulomatous manifestation (*n* = 26), and reported the survival time in this non-effusive FIP form as “up to 9 months” in a cat that was lost to follow-up. More careful retrospective record studies reported the survival time at 7–45 days in cats with histologically confirmed diagnosis (8 effusive, 5 non-effusive, no separate data provided; 9), and 7–60 days (*n* = 4, 1 of 4 was on IFNα-rHU; 10). Reports of clinical studies in natural infection put survival with dry FIP at 38 days (*n* = 1; 11), 1–171 days (*n* = 11; 6), 6–33 days without treatment (*n* = 4), and 4–42 days in cats previously given an FIP vaccine as a preventative (*n* = 4; 13).

The literature mentions an individual cat diagnosed with the dry form that survived for 200 days with glucocorticoid and ω-interferon treatment (6) and two cats survived 181 and 477 days with mixed dry/wet form treated with glucocorticoid, human α-interferon, and nelfinavir (7). A recent mention (18) of a natural survival time of over a year without treatment in cats with dry form FIP referred to cats that were treated with PI (15).

The survival times from this study were compared to published data of cats with dry form FIP. The data suggest a lengthening of the survival times in cats treated with PI. The survival times equaled or exceeded the longest survival times reported in the literature for dry FIP, with 8 of 60 cats in our study exceeding the maximum reported number of 200 days.

The diagnosis of dry FIP in our field study was done at different levels of diagnostic certainty, and we compared survival times in the subgroups—diagnosed, or not, by histologic, cytologic, or immunostaining tests and found no statistically significant differences based on the proof of the diagnosis, although there were significant differences between cats treated with corticosteroids concurrently with PI or not. There was no significant difference in the survival times of cats who did not receive steroids regardless of whether they were diagnosed by any biopsy-based method or diagnosed without invasive methods. The survival time was the same regardless of the diagnostic method used.

A comparison of mean and SD values for the survival times of cats with dry FIP showed that the survival time of cats in our study was significantly longer for the group treated with PI without concurrent corticosteroid treatment (201.4 ± 378.6 days, *n* = 27) than the published 38.4 ± 48.8 days (*n* = 11; 7); *p* = 0.04. The survival time for the group treated with corticosteroids concurrently with PI was 47.45 ± 49.26 days (*n* = 31), which does not significantly differ (*p* = 0.59) from the published value (7). Corticosteroids are the usual treatment for FIP signs; however, they need to be used with caution if PI is used.

Feline infectious peritonitis is considered 100% fatal which, in our study plan, precluded for ethical reasons an experimental design that used an untreated placebo group. For statistical comparison of data, a placebo group is ideal. This study was modeled on human studies that use single-arm trials of anti-cancer drugs with survival as the end-point. In those studies, no placebo or best accepted therapy is used because of the lack of known beneficial treatment and the universally grave prognosis (19). Our study was a field trial dependent on primary care veterinarians to identify candidates for the study and treat those accepted in accordance with the protocol. On ethical grounds and considering the reluctance of cat owners to participate in a placebo-controlled study, we elected the presented study design.

Our decision to exclude cats with wet form FIP was based on the rapid progression of the wet form (median of 9 days; 5) which we assumed correctly to be faster than the expected median time needed for acceptance of a cat into the study (which turned out to be 22.97 ± 21.60 days). Additionally, our prior limited studies of PI in effusive FIP did not appear promising while treatment of dry form FIP was encouraging.

The diagnosis of dry FIP was done at three levels of certainty: (1) highly probable, diagnosis supported by history, clinical signs, and laboratory findings without specialized lab tests; this category included neurologic forms in which biopsies are not possible and ocular forms when ocular centesis was used; (2) with histologic or cytologic confirmation of the diagnosis with or without immunostaining; this group also included cats with confirmation done on necropsied tissues; and its subgroup (3) with the confirmation of the presence of the FCoV antigen by IHC. There was no statistically significant difference in the survival between the cats diagnosed at different certainty levels and treated with PI only. We could not compare survival times between the subgroups treated with corticosteroids due to insufficient sample power.

Making an antemortem diagnosis of dry FIP is notoriously hard in the absence of a single, standardized test and relies on the combination of non-specific “disease-indicators” especially if the owners do not allow invasive procedures. Test data in clinical chemistry, hematology, serology, clinical assessment, histology, etc. have different predictive values and are not pathognomonic (2, 14, 18, 20). In the absence of a single, 100% reliable diagnostic approach, all diagnostic efforts are aimed to increase the “index of suspicion” as it was called by Diaz and Poma (21). The requirements for a “gold standard” vary between research groups with some accepting histopathology and others stressing IHC (14, 20), while the consensus document by Addie et al. (2) avoids the definition of the “definitive diagnosis” altogether. In clinical practice, most diagnoses are made by laboratory findings consistent with FIP and excluding other diseases (3, 18).

The primary care veterinarians and veterinary specialists treating these cats were comfortable with the diagnosis of dry form FIP, but we are using the term “presumptive FIP” because the “gold standard” of coronavirus antigen by IHC in lesions consistent with dry form FIP was not achieved with all cats. The cats in this study were diagnosed by their veterinarians based on the assessment of the combination of signalment, clinical signs, and as many tests as necessary to rule out other diseases and support the diagnosis, relying on experience (18), in the mode documented by Rohrbach et al. (22). The diagnosis, made by the primary care veterinarian in conjunction with specialists as needed, was reviewed by AML using criteria consistent with the evidence-based algorithm (2). Specialized testing such as histology, cytology, CSF tap, PCR on aqueous humor, IHC, and necropsy was done in 36/60 cats. When the presumptive diagnosis is made, owners are often reluctant to get the confirmation through the invasive procedures.

The diagnosis for each cat on the study was established individually, and we also compared our data to the data published for cats with a confirmed diagnosis of FIP (1, 7, 20, 23). Signalment and housing density were similar to the reported data (23) with the majority of the cats being young, non-pedigreed, and originating from multi-cat households. The age distribution of the FIP patients in our study underscored a well-established age bias for FIP (12, 22). Clinical presentations observed in our sample were similar to the reported for the cats with dry form FIP (20, 23).

Diseases that may be clinically similar to FIP were excluded by specific tests in most cats. Feline leukemia virus (FeLV) antigen and FIV antibody were measured in 50/60 cats and one of those was positive for FIV antibodies. The FIP diagnosis of the only FIV-positive cat was given by a neurologist based on the results of MRI and CSF tap. Antibodies for toxoplasma were measured in 18 cats and all were negative. Ten cats were not tested for FeLV and FIV; six had the diagnosis confirmed by specialized tests on the biopsied tissues (four IHC and two histology), and the other four had a number of strong FIP indicators and considered presumptive.

We analyzed the initial biochemical parameters of diagnostic value for FIP. In young cats, with high serum globulin and low A/G ratios, there are few conditions except FIP that are likely. Plasma cell and B-lymphocyte malignancies can produce a monoclonal gammopathy, but these conditions are rare in young cats. Chronic inflammatory or infectious conditions such as chronic abscesses and pyothorax could increase serum globulin levels but these conditions would likely be identified by diagnostic evaluation. Hyperglobulinemia and/or an albumin/globulin (A/G) ratio ≤0.6 were noted in 48 of the 50 (96%) cats; two cats had A/G ratio equal 0.8. At the initial evaluation of the cats in our study, there was hyperglobulinemia in 64.7% of the cats, which is similar to the value reported in the literature in the combined wet-dry group (7). The albumin/globulin ratio offers the highest positive prediction value for FIP (20). In our group it was 0.37 ± 0.14, which is consistent with the reported values for FIP in general (20) and lower than the mean and SD reported for the initial presentation by Tsai et al. (7).

Hyperbilirubinemia occurred in 16% of the cats which is consistent with dry form FIP and is lower than reported in the groups with wet FIP (7, 23). Hyperglobulinemia is an important diagnostic sign because it is rarely associated with diseases other than FIP in young kittens, which is the most often affected group.

The initial hematologic parameters of diagnostic value for FIP were analyzed. Leukocytosis and neutrophilia were found in 45.1 and 54.5% of the cats, respectively; both values are consistent with the published (23). Lymphopenia was present in 33.3% of the study cats consistent with previous studies (23).

Coronavirus antibody titers are somewhat helpful in the diagnosis of FIP (2, 4, 17). Negative serum antibody titers make the diagnosis of FIP unlikely, while high and very high titers are supportive of the diagnosis. However, mid-range to high antibody titers can occur with coronavirus infection without FIP. All tested cats accepted to the study had coronavirus antibody titers with most of the cats having high (400–1,280; *n* = 13), very high (>1,280, *n* = 36), or extremely high positive titers (>12,800, *n* = 10).

The only cat on the study without signs of FIP was diagnosed by the veterinarian after a pre-neuter checkup. The follow-up cytology of the mesenteric lymph node aspirate was inconclusive because the cellularity was insufficient to definitively demonstrate pyogranulomatous inflammation, although there was no evidence of neoplasia or other infectious organisms. The cat developed effusion and died after 148 days of the treatment with PI and corticosteroids concurrently.

The four cats who survived over 300 days had clinical signs and diagnostic tests consistent with FIP at initial examination. A detailed history and diagnostic information is given in Table 8 and Figures 5A–D. Although cats 52 and 78 did not have histopathology or IHC, their age (12 and 6 months), exposure in multi-cat, FCoV environments, clinical signs, laboratory findings, and high FCoV antibody titers were sufficient to establish a clinical diagnosis. Cats 2 and 105 had their diagnoses confirmed by histology and cytology and IHC. All four showed clinical improvement and returned to normal behavior. Cats 2 and 52 had only modest improvements in laboratory findings despite clinical improvements (Figures 5A,B). The clinical decline in those two cats started 2 weeks before their death and was accompanied by weight loss, anorexia, worsening anemia, reappearance of abdominal masses at the resection site (Cat 2), and pleural effusion in the same cat. Redevelopment of the abdominal mass and progression of the disease is common in the cats where the masses were resected (8).

The two longest survivors, cats 78 and 105, had weight loss at diagnosis but gained weight while on the PI treatment. The weight gain in cat 105 started 1 week before the trial and was attributed to prednisolone (5 mg daily). The prednisolone treatment was tapered and stopped during the first week of the PI treatment and the cat continued gaining weight. The clinical improvements and weight gain in both cats were accompanied by improvements in A/G ratios and hematocrits (Figures 5B,C). In both cats, PI treatment was either stopped or its frequency was decreased after over 2 years of survival. Cat 105 died of renal failure. Both cats declined at the end of life and were euthanized *in extremis*. The lack of pyogranulomatous changes in the biopsied tissues in cat 78 is consistent with the observation that cats experimentally infected with FIPV and who had clinical disease and survived were “free of lesions” post-mortem (14, 24). The necropsy changes of mild hepatic firbrosis and mild chronic lymphoplasmacytic interstitial nephritis are not adequate to account for the demise of the cat. There was no evidence of lesions of FIP. The A/G ratio of 0.5 at the initial diagnosis was returned to normal suggesting resolution of the FIP. No necropsy was performed on cat 105.

We did not use a formalized assessment of the quality of life. We collected information about clinical and behavior changes from progress reports, communications, and veterinarian charts. All progress reports and notes indicated an improved quality of life, returning to normal pre-diagnosis behavior as expressed in comments by both owners and veterinarians, e.g., “Very energetic … doing well,” “acts normal,” etc. In the medical records, cats were noted as having more energy, being more playful, interacting more with owners, and essentially returning to their pre-FIP behavior. In the 34 cats that lived for 30 + days, clinical improvement, i.e., an improvement in one or more signs such as increased appetite or an abatement of fever, was anecdotally noted after 10–14 days (four to six doses of PI); 10/34 reported weight gain, 5/34 reported weight loss, the weight remained stable for 17 cats, no records were filed for 2 cats. Generally speaking, the cats treated with PI returned to regular routines with occasional “bad days” until a precipitous decline led to death or euthanasia within days.

Our results suggest that PI benefits cats clinically diagnosed with dry FIP by increasing survival times and improving quality of life but a controlled study will be needed to verify the benefit of PI in the treatment of FIP. While not a cure, PI may maintain FIP cats as a chronic condition as opposed to the fast-progressing fatal disease. It may be possible to predict and monitor the survival by the normalization of A/G ratio and hematocrit. Survival times with PI treatment are significantly longer when corticosteroids are not used concurrently.

## Author Contributions

AL: conception and design of the work, interpretation of the data, revising the manuscript critically for important intellectual content, final approval of the version to be published. TK: post-study acquisition of the data, analysis and interpretation of data for the work, drafting the work and revising it critically for important intellectual content, final approval of the version to be published. GG: data acquisition, entry and organization, final approval of the version to be published. VB: data acquisition, entry and organization, revising the data and the manuscript critically for important intellectual content, final approval of the version to be published. RH: data analysis and interpretation, revising the manuscript critically for important intellectual content, final approval of the version to be published. All authors agree to be accountable for all aspects of the work in ensuring that questions related to the accuracy or integrity of any part of the work are appropriately investigated and resolved.

## Conflict of Interest Statement

AL, GG, VB, and RH do not have a financial interest in Sass & Sass, Inc. TK is an employee and a minor stakeholder in Sass & Sass, Inc. VB and RH were consultants to Sass & Sass. No financial incentives were provided to owners and veterinarians participating on the study.
